# Lifelong Glutathione Deficiency in Mice Increased Lifespan and Delayed Age-Related Motor Declines

**DOI:** 10.14336/AD.2024.1077

**Published:** 2025-11-22

**Authors:** J. Thomas Mock, Paapa Mensah-Kane, Delaney L. Davis, Jessica M. Wong, Philip H. Vann, Michael J. Forster, Nathalie Sumien

**Affiliations:** Department of Pharmacology & Neuroscience, University of North Texas Health Science Center, Fort Worth, TX 76107, USA

**Keywords:** glutathione, aging, oxidative stress, brain function

## Abstract

Glutathione (GSH) is a crucial redox scavenger, essential for maintaining cellular redox balance. This study explores the long-term effects of chronic GSH deficiency on lifespan, motor function, cognitive performance, redox status and inflammation. GCLM^-/-^ mice, with a 70-90% reduction in GSH levels, were compared to GCLM^+/+^ controls across their lifespan (5, 10 and 20 months). We assessed lifespan, motor performance using balance and coordination tests, cognitive function through anxiety and memory tests, redox markers, and inflammation markers, particularly TNF-α and IL-6. Biochemical analyses of GSH levels in peripheral tissues and brain regions were conducted to evaluate redox state changes. GCLM^-/-^ mice displayed extended lifespans and improved motor function at young and adult stages, with a delayed onset of motor decline with age. Cognitive function remains largely unaffected, although there are reductions in anxiety-related behaviors and minor deficits in fear-associated memory. Age-related increases in TNF-α, an inflammatory marker, are observed in both genotypes, with GCLM^-/-^ mice showing a less pronounced increase, particularly in females. There were significant GSH reductions in peripheral tissues, with sporadic changes in brain regions. This stress likely triggers compensatory antioxidant responses, modulating inflammation and redox-sensitive pathways. The data suggests that lifelong GSH deficiency provides protective effects against inflammation and motor decline in younger animals but exacerbates these issues in older mice. The study offers insights into potential therapeutic strategies that leverage mild oxidative stress to promote healthy aging, emphasizing the importance of redox state and antioxidant defenses in the aging process.

## INTRODUCTION

Aging is an inevitable and universal process characterized by the progressive deterioration of biological systems, resulting in a decline in functionality and an increased risk in mortality [1, 2]. This phenomenon is marked by escalating impairments in cognition, muscular strength, and motor function as well as increased susceptibility to chronic diseases [1, 3-5]. With an aging population in the United States, projected to double by the year 2060, there is a need to decipher the mechanisms associated with aging to identify potential targets to extend lifespan and healthspan [6]. The redox theory of aging postulates that as organisms age, there is a surge of reactive oxygen species (ROS) which causes a pro-oxidizing shift in reduction-oxidation (redox) state leading to cell signaling dysregulation, while moderate levels of ROS are a biologically useful signal [7]. Although several redox couples are responsible for the overall cellular redox state, glutathione (GSH) in the reduced or oxidized (GSSG) form is the predominant redox couple [8]. Therefore, modulation of redox state could be achieved by altering GSH and reduction in GSH has been implicated in aging alongside the GSH:GSSG ratio [8]. GSH is not only the main determinant of redox state but is also involved in cell signaling and cell regulation [9]. GSH synthesis is rate-limited by glutamate cysteine ligase (GCL), a heterodimer consisting of a catalytic (GCLC) subunit and a modifier (GCLM) subunit [9]. Elimination of the GCLC subunit is embryonic lethal, while genetic knockout of the GCL modifier subunit (GCLM^-/-)^ in mice leads to a 70-90% decrease in GSH levels across various tissues, including liver, brain, kidney, and lung [10]. Acute GSH deficiency has been shown to cause sustained impaired motor function and balance in young rodents, worsening motor neuron decline in disease models, impairment of cognitive function, and disruption of redox signaling in young and old rodents [11-14]. Research on the effects of lifelong redox dysregulation through systemic glutathione deficits is however limited and the long-term effects of glutathione depletion on lifespan, motor function, cognitive function, and overall redox state have not been addressed. Therefore, the goal of the current study was to determine the effects of chronic glutathione depletion on lifespan and healthspan by using a chronic model of GSH deficiency, GCLM^-/-^ mice [15]. Our working hypothesis was that lifelong glutathione depletion will exacerbate age-related declines in motor and cognitive function, decrease lifespan, and disrupt redox-sensitive proteins, due to limited defense against reactive oxygen species and disruption of redox status and redox signaling. The outcomes of the study are important in determining the impact of glutathione on the aging process and exploration of interventions modulating GSH levels to mitigate the effects of aging.

## MATERIALS AND METHODS

### Animals

All procedures adhered to NIH guidelines and were approved by the Institutional Animal Care and Use Committee at the University of North Texas Health Science Center. Heterozygous GCLM (GCLM^+/-^) mice (generated on a C57BL/6J (B6.129)) background were obtained from Dr. Terrance Kavanagh from University of Washington, and rederived and backcrossed for at least 7 generations into C57BL/6 mice by Jackson Laboratories. Mice were then maintained in the UNT HSC vivarium and GCLM^+/-^ triads were mated to obtain wild-type (GCLM^+/+^) and knock-out (GCLM^-/-^) mice. From our in-house breeding colony, female and male mice were housed in standard polycarbonate cages in groups of 2-4 (separated by sex and genotype) and had ad libitum access to standard rodent chow (LabDiet® R&M 5LG6 5S84; catalog number: 1813505 from TestDiet, Richmond, IN) and water, at 23 ± 1° C, under a 12 h light/dark cycle starting at 0600. Animals were identified via a subcutaneously embedded Rf scannable chip.

**Figure 1.**
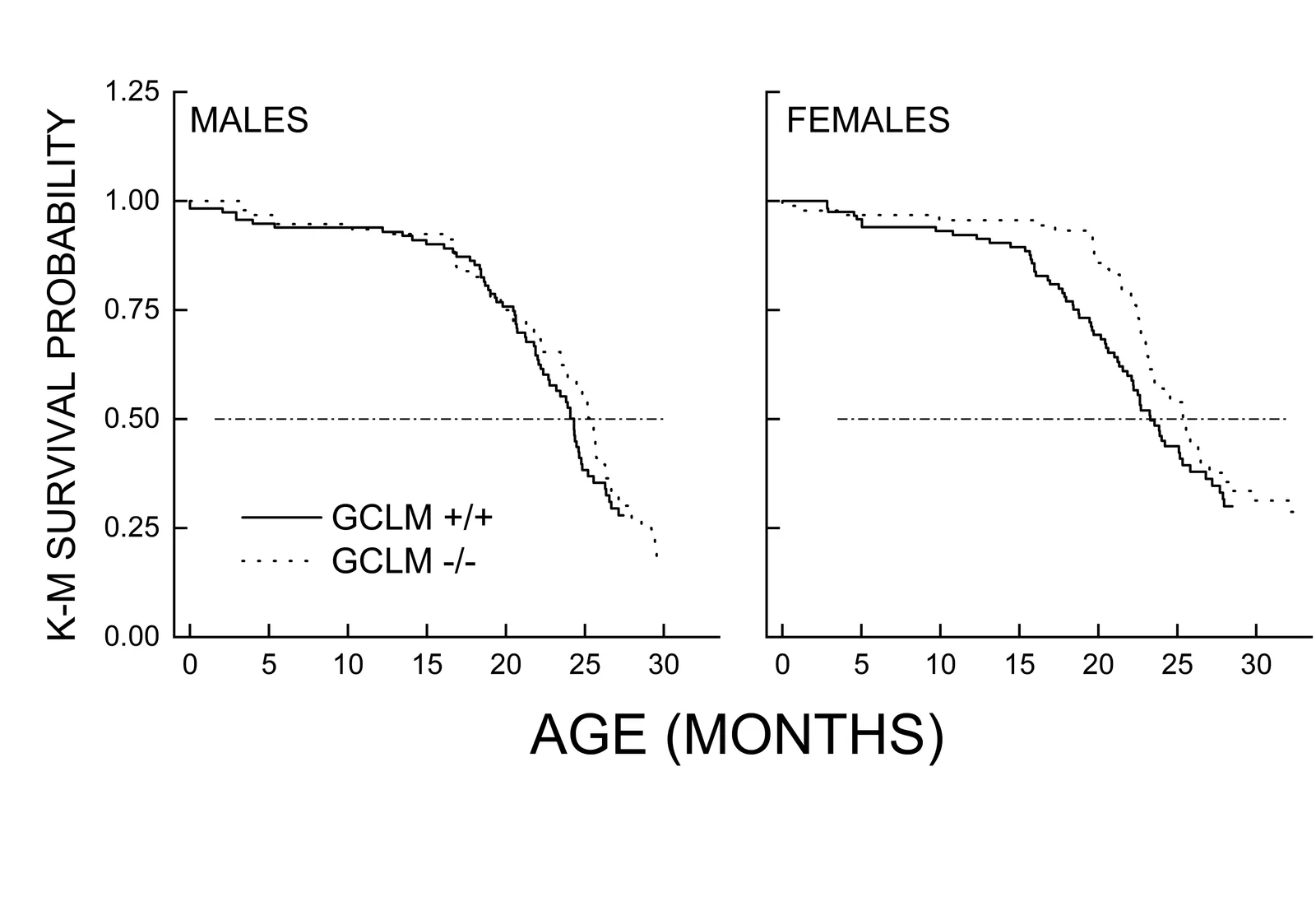
**Effects of Sex and Genotype on Kaplan-Meier (K-M) survival probability**. Gclm^-/-^ started with 93-94 mice and Gclm^+/+^ started with 115-118 mice (total of 420 mice included in the lifespan study.

A total of 420 mice were used in the lifespan analyses of the two genotypes. All mice were used in the survival analyses. If they died of natural causes, they were counted as exact failures while the ones that were euthanized after behavior were right censored (Fig. 1). From the colony, mice at the target ages were then randomly assigned to behavioral cohorts (others were assigned to tissue collection only). Once randomly assigned to an experimental group and the mice were behaviorally tested at 5, 10, or 20 months (we started with 62 young, 63 middle-age, and 75 old mice with an n of 15-21 mice/sex/genotype). The behavioral tests were conducted in a blinding fashion with the tester not being given the genotype of the mice until behavioral tests were completed. Data analyses were checked by independent researchers. For the cohorts used for behavior and biochemical analyses, weights were done weekly during testing, and food intake was measured the week prior to the start of the behavioral battery. All behavioral tests were conducted on the same mice. The tests were spaced out to minimize fatigue and potential bias, ensuring that multiple behavioral tests on the same mouse were performed with adequate recovery time between tests. For food intake, cages were given a set amount of food and daily measurements for 4 days were taken and averaged to estimate food consumption of the mice.

### Neurobehavioral Testing

Elevated Plus Maze- Mice were placed in the center of the maze and given 5 min to explore. Using a tracking system (ANY-MAZE, Stoelting), time spent in open arms and distance traveled were analyzed.

Locomotor Activity- In a sound-attenuating chamber equipped with a fan (white noise set at 80dB), mice were given 16 minutes to explore the test cage (surrounded by photocells; Digiscan apparatus, Omnitech Electronics, Columbus OH). Horizontal, vertical and spatial components were processed by a software program (Fusion v5.5 Superflex Edition, Omnitech Electronics, Columbus, OH).

Walk Initiation- The time taken for a mouse to move one body length was recorded daily and averaged over four consecutive days.

Alley Turn- The time taken for a mouse to completely turn around in black alleyway when facing the back wall was recorded daily and averaged over four consecutive days.

Negative Geotaxis- The time taken for a mouse to turn 90° on a 45° inclined wire mesh platform was recorded daily and averaged over four consecutive days.

Wire Suspension- Each mouse was allowed to grip a horizontal wire suspended 27 cm above a padded surface. The latency to tread (grasp the wire with their hind legs) and the latency to fall were recorded and averaged over four consecutive daily sessions (2 trials/day).

Bridge Walking- The latency to fall (up to a maximum of 60 s) from a bridge mounted 50cm above a padded surface was measured daily (3 trials/day) and averaged over four consecutive days. The bridges increased in difficulty daily (side (small: 1 cm or large: 2 cm) and shape (round or square)). The mouse was placed on one of the platforms for 5 s and then gently dragged to the middle of the bridge in which the mice had to traverse to one of the platforms.

Coordinated Running- Using an accelerating rotorod (Accuscan Instruments, Model #AIO501RRT527M; AIO411RRT525M), latency to fall from the rod was measured. On a given trial, mice were placed on the cylinder that rotated with an acceleration of 0 to 75 rpm in 150 seconds. Acceleration continued until either 75 rpm is reached or the last animal had fallen to the padded surface. Each session consisted of four trials and mice received two sessions daily for at least seven sessions. After the seventh session, performance stability was calculated and tested until they reached a “stability criterion” of three consecutive sessions over which the four-trial mean latency to fall did not differ by more than 15%.

Spatial Learning and Memory- Straight Alley-In the pre-training phase, a black curtain was positioned above a tank filled with opacified water maintained at 24±10 C to minimize visual cues. Each mouse was placed at the end of the straight alley (10 x 65 cm) and must reach the hidden platform at the opposite end within 60 s. The mice underwent 2 sessions (Friday and Monday) and each session consisted of 5 trials with an intertrial interval of 5 minutes (data not presented). Place Discrimination Acquisition-The curtain and straight alley were removed, and the mice were tested on their ability to use distal cues to locate the hidden platform in the tank. The mouse was lowered in the water at one of four starting locations and was given 90 s to find the platform. Each daily session consisted of five platform trials (with 90s ITI) for a total of 9 sessions (with a weekend break between sessions 4 and 5). The measures of performance for this phase were the distance traveled and latency to reach the platform and speed. In sessions 2, 4, 5, 7, and 9, a probe trial was conducted as the 5th trial instead of a platform trial. During the probe trial, the platform was inaccessible to the mice for 30 s and once the trial ended, the platform was raised, and the mice were given 60 s to find the platform. For probe trials, spatial bias for the location of the platform was recorded in terms of the percentage of time spent within 40-cm diameter annuli surrounding the platform location. Visible Platform- Ten sessions were administered, with each session consisting of four trials at 10-min intervals (2 sessions/day). On each trial, the mouse was placed in a different starting point and had to swim to the platform which was also moved to a different location. Performance across sessions was measured as path length taken to reach the platform. A computerized tracking system (ANY-maze, Stoelting, Chicago, IL) was used to record the position of the mice.

Forced swim test- The test consisted of one trial in which the mouse was placed in the tank filled with water (24±1°C) in which they can swim freely but cannot escape for a total of 6 min. The immobility time after the first 2 min was recorded using a stopwatch.

Auditory Startle Response- Each mouse was placed inside an acrylic cylinder and presented with a series of mixed-frequency bursts of auditory stimuli (0, 90, 100, 110, 120 or 140 dB). Each acoustic signal lasted 20 ms and was presented 12 times (in a counterbalanced series), for 72 trials. The amplitude of the startle reflex was characterized as the peak response to each auditory intensity (SA Lab, San Diego Instruments).

Discriminated Avoidance- During acquisition, an initial preference trial was given in which a scrambled shock (0.69mA) was initiated and then terminated upon entry by the mouse into either goal arm. The correct goal arm was then defined as the arm opposite than the arm the mouse initially chose. On subsequent trials, shock was initiated 5 s after either opening the start box door or immediately upon an entry into the incorrect goal arm and was terminated after entry into the correct goal arm (maximum time of 60 s). Trials continued at 60 s intervals until the animal reached criterion (choice of correct arm under 5 s in four out of the five consecutive trials) or 25 trials had elapsed. Upon completion of the acquisition phase, mice were returned to their home cage for 1 h and then underwent the first reversal session. The test was conducted the same way as the acquisition phase, except the correct arm was now designated the opposite arm based on the previous session. The second reversal session started 1 h after the completion of the first reversal session. The performance of the mice was measured as the number of trials taken to reach criterion across the 3 sessions.

Fear Conditioning- Day 1 was the conditioning test in which the mouse was placed in an unfamiliar environment (conditioning context) consisting of a grid floor and white walls with black, vertical stripes. In a 5-min session, the mouse was presented with 2 pairings of a loud sound (conditioned stimulus; 2000 Hz) and a brief foot shock (unconditioned stimulus; 0.69 mA for 2 s). After 24 hours the mice returned to the now familiar context in a 5-min session with no conditioned or unconditioned stimulus (context conditioning test). One hour after the context conditioning test, was the novel context test in which the mouse was placed in a novel environment consisting of a grey, smooth floor and grey walls for 3-min. The conditioned stimulus test occurred immediately after the context conditioning test in which the conditioned stimulus was present for 3-min. Percentage of time freezing was recorded by ANY-maze (Stoelting, Chicago, IL) for analysis.

Optomotor Task- A computer program (OptoMotry, CerebralMechanics, Lethbridge, Alberta, Canada) projected visual stimuli (vertical gratings) onto the platform-surrounding computer monitors. The gratings rotated at 12°/s and visual acuity threshold was determined by projecting a low spatial frequency grating (0.042 cycles/degree) onto the four walls and rotating either clockwise (testing the left eye) or counterclockwise direction (testing the right eye) and set at the highest spatial frequency to which the animal produced a response. A staircase method was implemented to determine visual acuity threshold through a series of gratings of that increased with higher spatial frequencies (rotating in one direction and then the opposite) for as long as the mouse could detect the grating movements.

### Biochemical Measurements

At the target ages of 5, 10 or 20 months, tail bleeding was performed on the mice just prior to euthanization (cervical dislocation). Blood was collected in a tube containing EDTA and spun at 5,000G for 10 min. Brains were dissected into three regions (cerebral cortex, cerebellum, striatum), liver and skeletal muscle were collected. All tissues were snap frozen in liquid nitrogen and stored at -80°C until further processing.

Redox State- Samples for redox state measurement were pulverized and homogenized in liquid nitrogen via mortar and pestle and shipped frozen on dry ice to the Oklahoma Nathan Shock Center - Redox Biology Core for determination of redox state measures. Levels of GSH, NADH, and NADP in cerebellum, cortex, striatum, and skeletal muscle were quantified using reverse-phase HPLC and electrochemical detection. Proteins were precipitated upon incubation on ice (5 min) followed by centrifugation (10 min at 16,000 g). The supernatant was filtered (0.45-μm syringe filters) and metabolites were resolved by HPLC and quantified by electrochemical detection (Shimadzu HPLC system, ESA Coularray electrochemical detector 5600A set at 750 mV). Metabolites were eluted through a C18 column (Phenomenex Luna C18(2), 100 Å, 3 μm, 150×4.6 mm) at 0.5 ml/min using an isocratic mobile phase consisting of 25 mM NaH2PO4, 0.5 mM 1-octane sulfonic acid, 4% acetonitrile, pH 2.7. Metabolite concentrations were calculated by employing standard curves constructed from peak areas.

Plasma Inflammatory Markers- Commercial sandwich-based enzyme linked immunosorbent assays (ELISA) were used to determine plasma IL6 and TNF-α (R&D Quantikine). Samples were added to a pre-coated 96 well plate, incubated for 2 hours, washed 5X, incubated with conjugate for 2 hours, washed 5X, incubated with substrate solution for 30 min, and then the reaction was stopped via acid and read within 30 min at 540 nm.

### Statistical Analysis

Data analysis of the data involved three-way analyses of variance (ANOVA) with Sex, Age, and Genotype as between group factors for each independent measure using the car package in R. Furthermore, variables from behavioral tests with multiple sessions were analyzed with repeated measures three-way ANOVA (with Session, Week, or Month as the within group factor). Following significant main effects or interactions, planned individual comparisons between different age groups (5, 10, or 20 months), sex (male or female), and genotype (GCLM^+/+^ and GCLM^-/-^) were performed using a single degree-of-freedom F tests involving the error term from the overall ANOVA. Data analyses of the food intake, redox and inflammatory markers were done using a non-parametric test, Kruskal-Wallis with Group as a factor. Following significant effect of Group, Conover-Inman pairwise comparisons between groups were performed. Kaplan-Meier survival distributions were calculated and log-rank Chi-Quare statistics (Tarone-Ware) were used to compare mortality rates among the two genotypes for males and females separately. For all analyses, R 3.5 (R Foundation, Vienna, Austria) or Systat 13 were used and the α level was set at 0.05.

**Figure 2.**
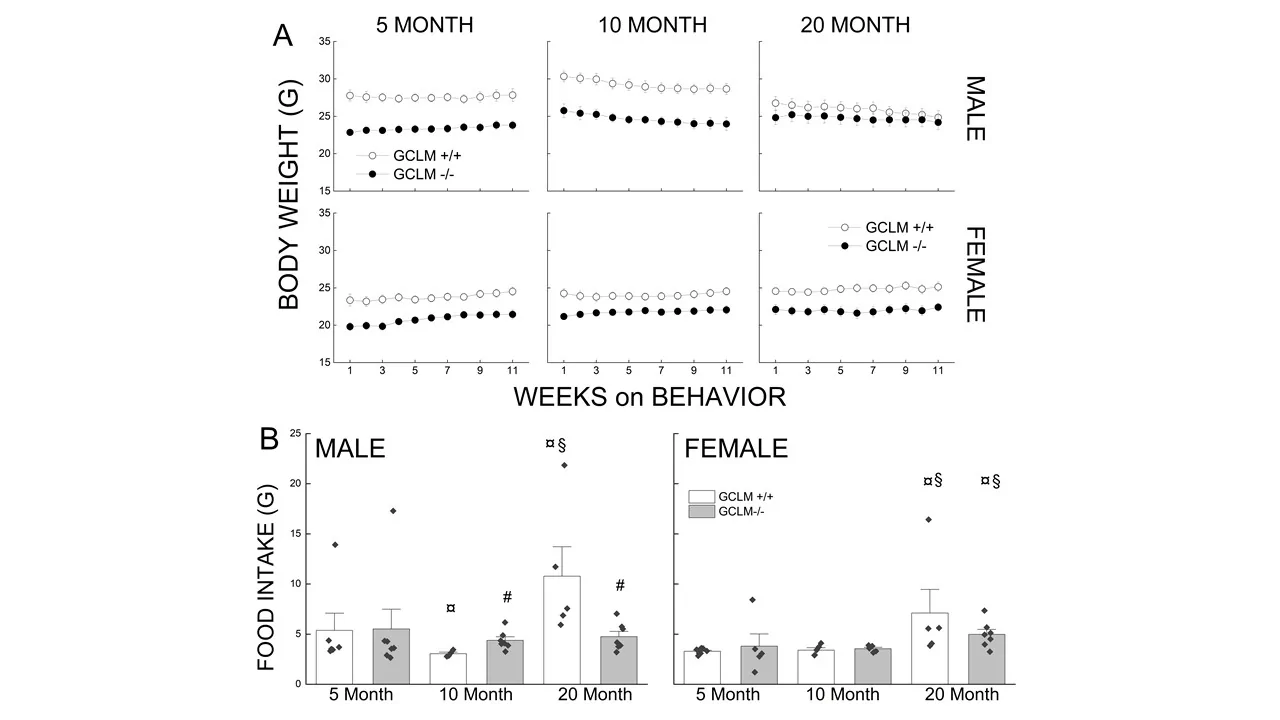
**Effects of Sex, Age, and Genotype on body weight and food intake**. Effects of Sex, Age, and Genotype on body weight (A) and food intake (B) during behavior in young (5 month), adult (10 month), and old (20 month) GCLM^+/+^ and GCLM^-/-^ mice. Each value represents the mean ± SEM (n = 13-24 for body weights, n=4-7 for food intake). Post-hoc analyses: +*p* < 0.05 compared to age and genotype-matched males; ¤*p* < 0.05 compared to genotype and sex-matched young; §*p* < 0.05 compared to genotype and sex-matched adult; #*p* < 0.05 compared to age and sex-matched GCLM^+/+^ mice.

## RESULTS

Survival Analysis -The effects of Sex and Genotype on Kaplan-Meier probabilities of survival time are presented in Fig. 1. In female GCLM^-/-^ mice there was a delay in initial mortality compared to female GCLM^+/+^, while there did not seem to be any effect in the males. Tarone-Ware Chi-square analyses revealed a significant effect of genotype in the females (*p*=0.042) but not in the males (*p*=0.683).

Body Weight and Food Intake-The effects of Sex, Age, and Genotype on body weight during behavior are presented in Fig. 2A. Overall, GCLM^-/-^ mice weighed less, and males weighed more than females regardless of age or genotype. A three-way repeated measures ANOVA revealed significant main effects of Sex (p < 0.001), Age (p < 0.05), and Genotype (p < 0.001), but no within-group effect of Week (p = 0.95). There were significant interactions of Sex*Age (p < 0.01), Age*Genotype (p < 0.05), Sex*Week (p < 0.001) and Age*Week (p < 0.001), but no other interactions (all ps > 0.5).

**Figure 3.**
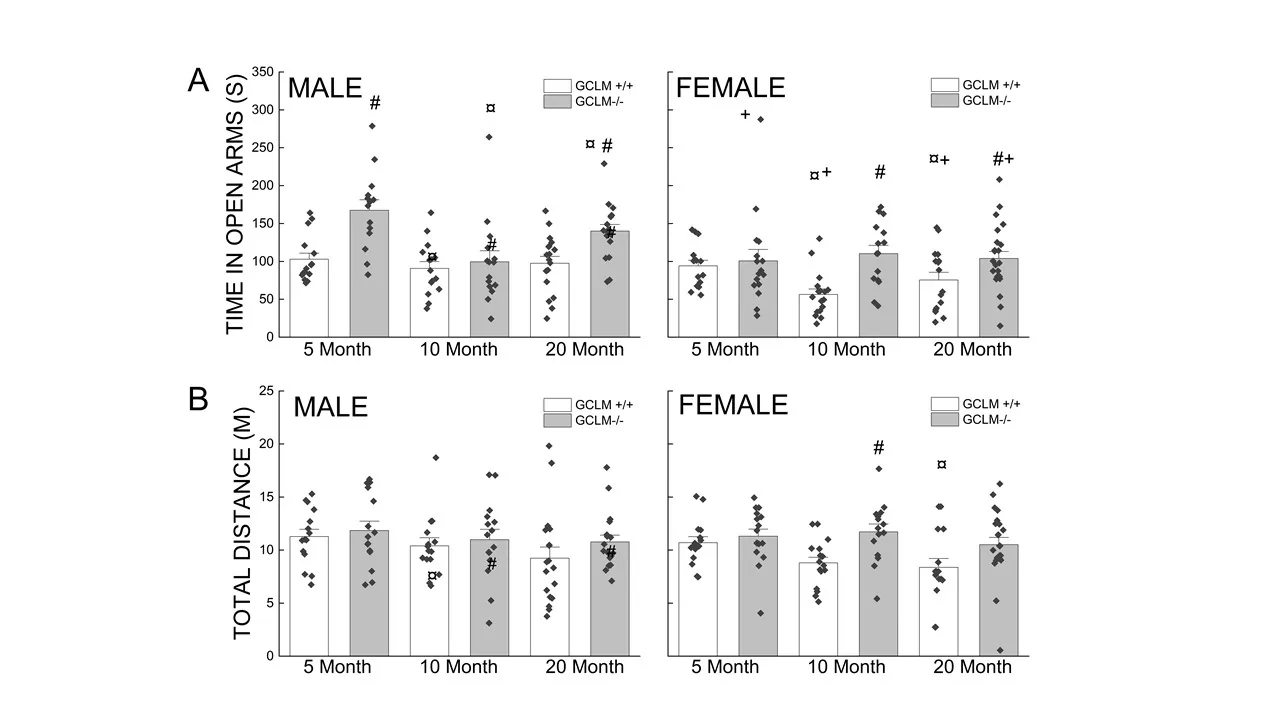
**Effects of Sex, Age and Genotype on elevated plus maze test performance**. Effects of Sex, Age and Genotype on time spent in the open arms (A) and distance traveled (B) during the elevated plus maze test in young (5 month), adult (10 month), and old (20 month) GCLM^+/+^ and GCLM^-/-^ mice. Each value represents the mean ± SEM (n = 15-23). Post-hoc analyses: +*p* < 0.05 compared to age and genotype-matched males; ¤*p* < 0.05 compared to genotype and sex-matched young; #*p* < 0.05 compared to age and sex-matched GCLM^+/+^ mice.

The effects of Sex, Age, and Genotype on food intake just prior to behavioral testing are presented in Fig. 2B. Non-parametric analyses revealed a main effect of Group, with overall minor effects of genotype at 10 and 20 months in the males and minor effects of age for both sexes. Some of these effects might be due to food wasting rather than true differences.

Elevated Plus Maze (anxiety-like behavior)- The effects of Sex, Age, and Genotype on time spent in the open arms are presented in Figure 3A. In GCLM^+/+^ groups, there were no differences at different ages in the males, but 10- and 20-month females spent less time in the open arms compared to the 5-month group. In GCLM^-/-^ groups, the 10- and 20-month males spent less time in the open arms compared to the 5-month group, while no effect of age was observed in females. GCLM^-/-^ mice spent more time in open arms than the age-matched GCLM^+/+^ at 5 and 20 months in males and at 10 and 20 months in females. A three-way ANOVA revealed significant main effects of Sex (p < 0.0001), Age (p < 0.01), and Genotype (p < 0.0001), and an interaction between Sex, Age, and Genotype (p < 0.01).

The effects of Sex, Age, and Genotype on total distance traveled are presented in Figure 3B. A moderate decline in total distance was observed across age in GCLM^+/+^ mice, especially in females, while there was no age-related decline in GCLM^-/-^. GCLM^-/-^ traveled as far or more than age- and sex-matched GCLM^+/+^ mice, notably in 10- and 20-month females. A three-way ANOVA revealed a significant main effect of Age (p = 0.02) and Genotype (p < 0.01), but no interactions between Sex, Age, and Genotype (all ps > 0.25).

**Figure 4.**
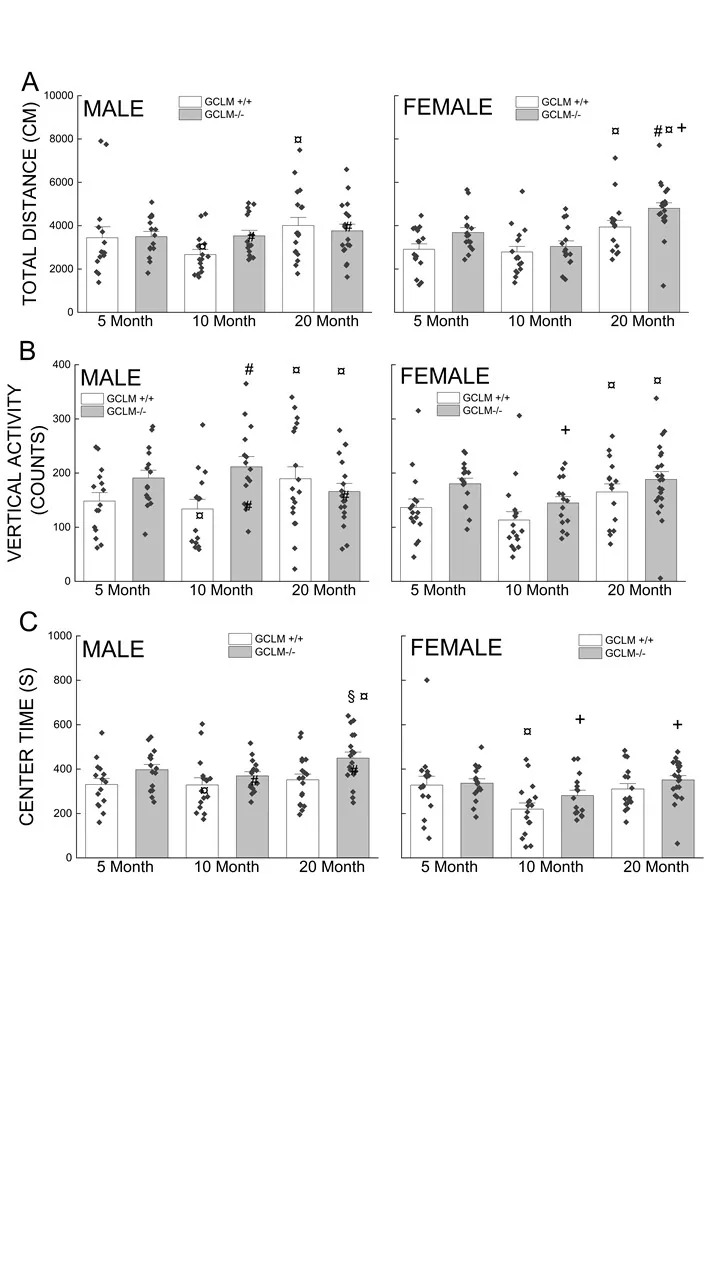
**Effects of Sex, Age and Genotype on spontaneous activity**. Effects of Sex, Age and Genotype on total distance traveled (A), vertical activity (B) and time spent in the center (C) during the spontaneous locomotor activity test in young (5 month), adult (10 month), and old (20 month) GCLM^+/+^ and GCLM^-/-^ mice. Each value represents the mean ± SEM (n = 15-22). Post-hoc analyses: +*p* < 0.05 compared to age and genotype-matched males; ¤*p* < 0.05 compared to genotype and sex-matched young; §*p* <0.05 compared to genotype and sex-matched adult; #*p* < 0.05 compared to age and sex-matched

Locomotor Activity (Spontaneous activity measure)- The effects of Sex, Age, and Genotype on total distance traveled in the open field are presented in Fig. 4A. In males, distance travelled was higher in 20-month-old GCLM^+/+^ compared to 10-month-old GCLM^+/+^. In females, total distance travelled increased in 20-month-old GCLM^-/-^ mice when compared to 5 months, and to a greater extent than in 20-month-old GCLM^+/+^ mice. A three-way ANOVA revealed significant main effects of Age (p < 0.0001), and Genotype (p = 0.01), but no interactions between Sex, Age, and Genotype (all ps > 0.10). Vertical activity (Fig. 4B.) was higher in 20-month-old GCLM^+/+^ mice compared to the 5- and 10-month-old age groups. In GCLM^-/-^, vertical activity increased only in 20-month-old females compared to 10 months, and to a greater extent than in 20-month-old GCLM^+/+^ females. A three-way ANOVA revealed significant main effects of Sex (p = 0.05) and Genotype (p < 0.001) and approached significance for Age (p = 0.06). There was a significant Age*Genotype interaction (p = 0.03), but no other interactions (all ps > 0.11). Time spent in the center zone (Fig. 4C.) decreased in 10-month-old GCLM^+/+^ females and increased by 20 months, while there was no change with age in males. Overall, GCLM^-/-^ males spent more time in the center compared to GCLM^+/+^ males. A three-way ANOVA revealed a significant main effect of Sex (p < 0.01), and interactions between Sex*Genotype (p = 0.02) and Age*Genotype (p = 0.03). Although an interaction of Sex*Age*Genotype approached significance (p = 0.08) there was no interaction of Sex*Age (p = 0.99).

**Figure 5.**
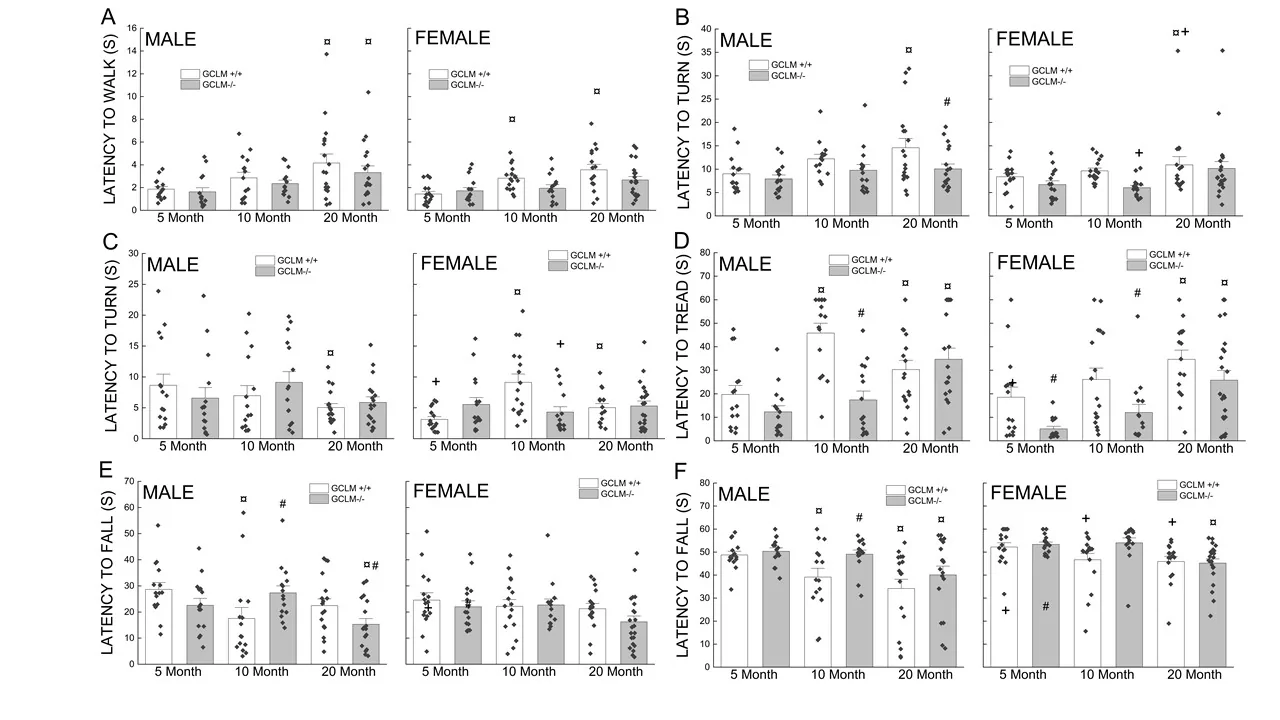
**Effects of Sex, Age and Genotype on reflexes, strength and balance**. Effects of Sex, Age and Genotype on walk initiation (A), alley turn (B), negative geotaxis (C), wire suspension (D: time to tread; E: time to fall) and bridge-walking (F) in young (5 month), adult (10 month), and old (20 month) GCLM^+/+^ and GCLM^-/-^ mice. Each value represents the mean ± SEM (n = 15-22). Post-hoc analyses: +*p* < 0.05 compared to age and genotype-matched males; ¤*p* < 0.05 compared to genotype and sex-matched young; #*p* < 0.05 compared to age and sex-matched GCLM^+/+^ mice.

Walk Initiation (reflex measurement)- The effects of Sex, Age, and Genotype on walk initiation are presented in Fig. 5A. Latency to walk was higher at 10 and 20 months in GCLM^+/+^ mice compared to 5 months. In GCLM^-/^, age-related changes were smaller than in the GCLM^+/+^ and only significant at 20 months in males. A three-way ANOVA revealed significant main effects of Age (p < 0.0001) and Genotype (p = 0.04), but there was no main effect of Sex (p = 0.19) or interactions (all ps > 0.31).

Alley Turn (reflex measurement)- In this test, presented in Fig. 5B, GCLM^+/+^ mice, had a higher latency to turn at 10 and 20 months compared to 5 months, though the age effect was larger in males. There were no major changes with age in GCLM^-/-^ male mice, while an age effect was apparent in 20-month-old females. A three-way ANOVA revealed significant main effects of Sex (p < 0.01), Age (p < 0.001), and Genotype (p < 0.01), but no other interactions (all ps > 0.28).

**Table 1:** Behavioral Measures.

	5 MONTHS				10 MONTHS				20 MONTHS			
	MALE		FEMALE		MALE		FEMALE		MALE		FEMALE	
	GCLM^+/+^	GCLM^-/-^	GCLM^+/+^	GCLM^-/-^	GCLM^+/+^	GCLM^-/-^	GCLM^+/+^	GCLM^-/-^	GCLM^+/+^	GCLM^-/-^	GCLM^+/+^	GCLM^-/-^
% TIME IN AN40	31.1±2.2	29.2±2.0	31.9±2.0	26.6±1.9	24.4±1.8¤	27.1±1.5	28.9±2.9	26.5±2.2	26.7±2.3	24.1±1.9	23.6±1.9¤	21.0±1.8¤
VISIBLE PLATFORM	116.2±10.0	113.5±5.3	119.2±18.1	102.1±6.6	112.0±5.7	110.1±6.8	116.5±9.1	116.8±6.1	114.8±4.4	112.7±6.8	126.8±9.5	107.7±6.0
VISUAL ACUITY	0.40±0.02	0.33±0.02	0.39±0.02	0.43±0.03+	nt	nt	nt	nt	0.35±0.01	0.32±0.01	0.33±0.03¤	0.31±0.01¤

Post-hoc analyses: +*p* < 0.05 compared to age and genotype-matched males; ¤*p* < 0.05 compared to genotype and sex-matched young; §*p* < 0.05 compared to genotype and sex-matched adult; #*p* < 0.05 compared to age and sex-matched GCLM^+/+^ mice. nt: not tested. AN40 (Annulus 40): n = 13-18; Visible Platform: n = 13-17; Visual Acuity: n = 7-15.

Rotorod (coordinated running and motor learning Negative geotaxis (reflex measurement)- The latency to turn 90° in the negative geotaxis test (Fig. 5C) declined between 10 and 20 months in GCLM^+/+^ males but had the opposite in females. In GCLM^-/-^ mice, there were no age-related effects observed. A three-way ANOVA revealed significant main effects of Sex (p = 0.02) and Age (p = 0.04), but there was no main effect of Genotype (p = 0.77), and the only interaction was between Sex*Age*Genotype (p < 0.01).

**Figure 6.**
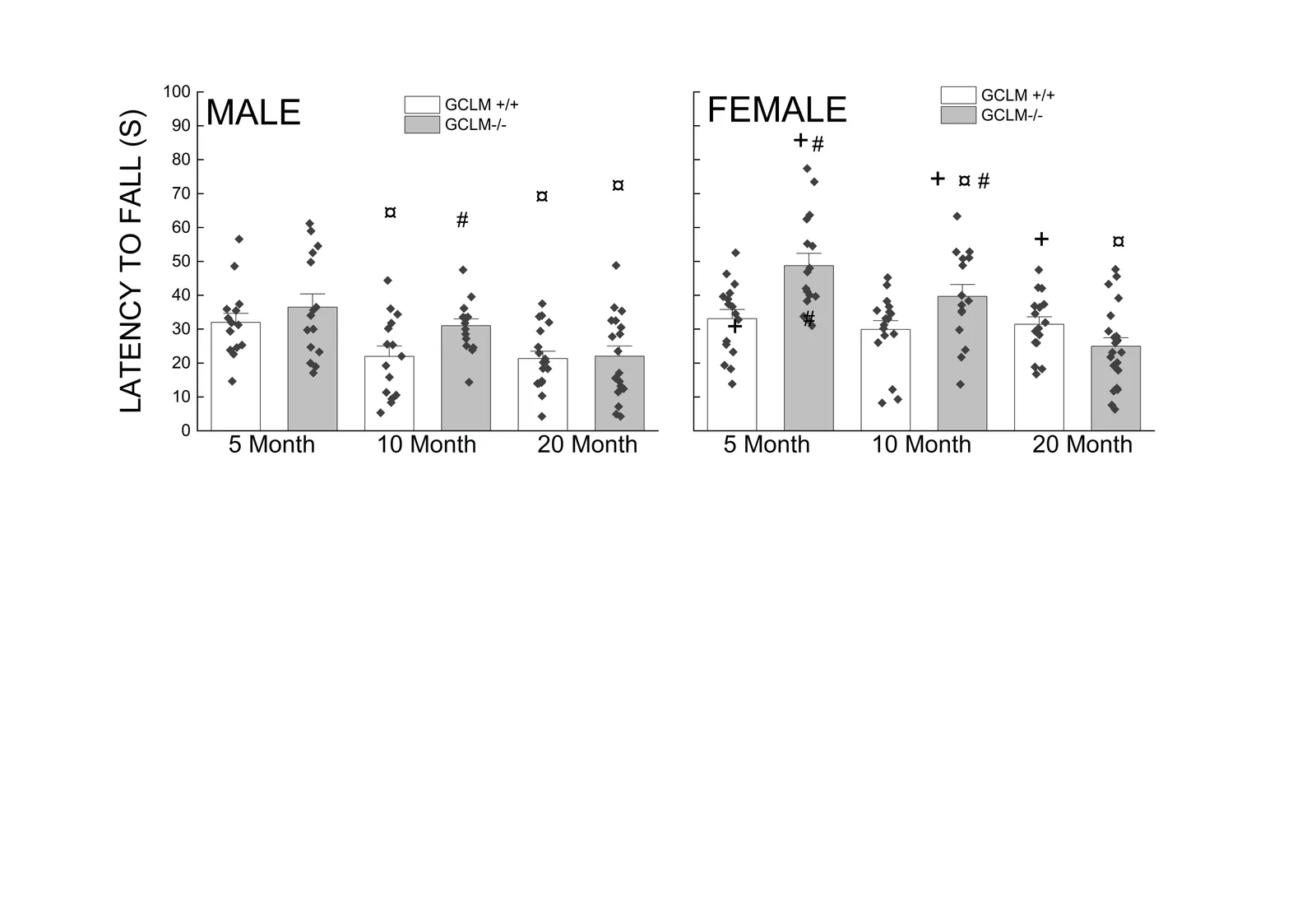
**Effects of Sex, Age and Genotype on coordinated running performance**. Effects of Sex, Age and Genotype on latency to fall from the rotorod in young (5 month), adult (10 month), and old (20 month) GCLM^+/+^ and GCLM^-/-^ mice. Each value represents the mean ± SEM (n = 15-22). Post-hoc analyses: +*p* < 0.05 compared to age and genotype-matched males; ¤*p* < 0.05 compared to genotype and sex-matched young; #*p* < 0.05 compared to age and sex-matched GCLM^+/+^ mice.

Wire Suspension (reflex and strength measurement)- The effects of Sex, Age, and Genotype on latency to tread are presented in Fig. 5D. In GCLM^+/+^ mice, latency to tread increased in males between 5 and 10 months and gradually increased between 10 and 20 months for females. While GCLM^-/-^ mice also had age-related changes, it was mainly between 10 and 20 months for both sexes. GCLM^-/-^ groups had shorter latencies to tread than GCLM^+/+^ at 5 and 10 months, but there was no difference between the two genotypes at 20 months. A three-way ANOVA revealed significant main effects of Sex (p < 0.01), Age (p < 0.0001) and Genotype (p < 0.0001) with interactions of Age*Genotype (p < 0.01) and Sex* Age*Genotype (p = 0.04). The effects of Sex, Age, and Genotype on latency to fall from a wire are presented in Fig. 5E. In GCLM^+/+^ mice, latency to fall from a wire (Fig. 5E) decreased mainly between 5 and 10 months in males, while there were no changes in females. In GCLM-/- mice, latency to fall was decreased mostly between 10 and 20 months for both sexes. These observations were supported by a three-way ANOVA that revealed a significant main effect of Age (p < 0.01) and an interaction of Age*Genotype (p < 0.01) but there were no other main effects or interactions (p > 0.18). A three-way ANCOVA revealed significant main effects of Age (p < 0.01) and Weight (p < 0.05) and a significant interaction for Age*Genotype (p = 0.01), but there were no other main effects or interactions (all ps > 0.17).

**Figure 7.**
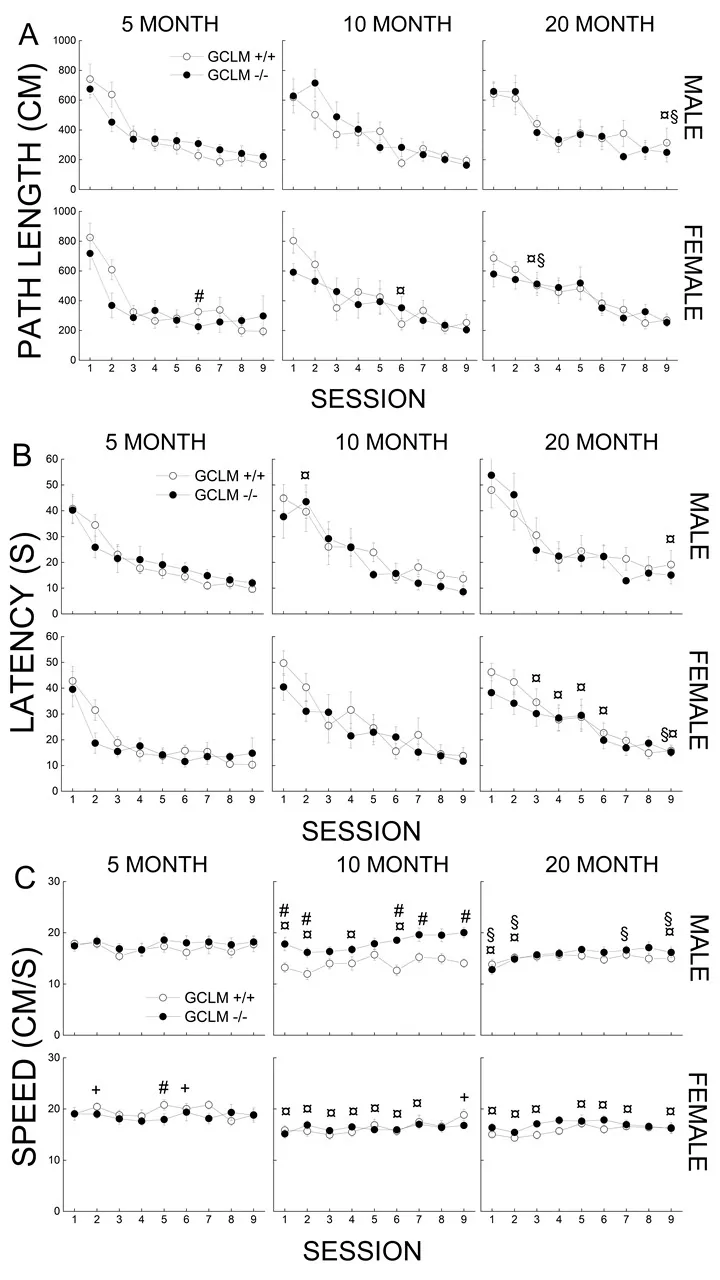
**Effects of Sex, Age and Genotype on Morris water maze performance**. Effects of Sex, Age and Genotype on distance (A), time to platform (B) and swim speed (C) in young (5 month), adult (10 month), and old (20 month) GCLM^+/+^ and GCLM^-/-^ mice. Each value represents the mean ± SEM (13-15). Post-hoc analyses: +*p* < 0.05 compared to age and genotype-matched males; ¤*p* < 0.05 compared to genotype and sex-matched young; §*p* < 0.05 compared to genotype and sex-matched adult; #*p* < 0.05 compared to age and sex-matched GCLM^+/+^ mice.

Bridge Walking (balance measurement)- The effects of Sex, Age, and Genotype on overall average latency to fall from an elevated bridge are presented in Fig. 5F. In GCLM^+/+^ mice, latency to fall decreased mainly between 5 and 10 months, and to a greater extent in males. In GCLM^-/-^ mice, latency to fall decreased between 10 and 20 months in both sexes. A three-way ANOVA revealed significant main effects of Sex (p < 0.001), Age (p < 0.0001) and Genotype (p < 0.01), but there were no interactions between Sex, Age, or Genotype (all ps > 0.12).

**Figure 8.**
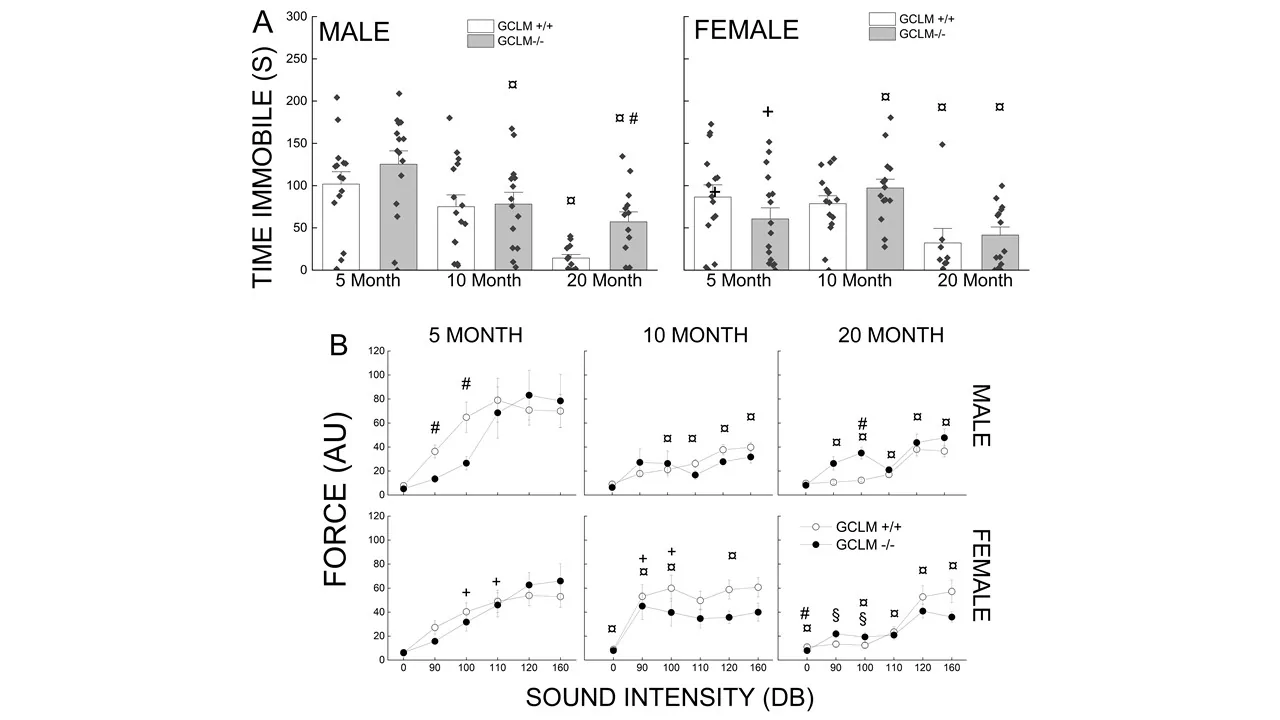
**Effects of Sex, Age and Genotype on forced swim test performance and audition**. Effects of Sex, Age and Genotype on immobility in the forced swim test (A) and auditory startle response (B) in young (5 month), adult (10 month), and old (20 month) GCLM^+/+^ and GCLM^-/-^ mice. Each value represents the mean ± SEM (n = 13-15). Post-hoc analyses: +*p* < 0.05 compared to age and genotype-matched males; ¤*p* < 0.05 compared to genotype and sex-matched young; §*p* < 0.05 compared to genotype and sex-matched adult; #*p* < 0.05 compared to age and sex-matched GCLM^+/+^ mice.

Rotorod (coordinated running and motor learning measurement)- The effects of Sex, Age, and Genotype on the latency to fall from a rotating rod are presented in Fig. 6. In GCLM^+/+^ mice, latency to fall decreased with age in males at 10 and 20 months, with no age-related decline in females. In GCLM^-/-^ mice, latency to fall decreased at 10 and 20 months, but to a lesser extent than 10-month-old GCLM^+/+^ mice. A three-way ANOVA revealed significant main effects of Sex (p < 0.0001), Age (p < 0.0001) and Genotype (ps < 0.01), and a significant interaction of Age*Genotype (p < 0.01). Although an interaction of Sex*Age*Genotype approached significance (p = 0.08), there were no other interactions (all ps > 0.62).

Morris water maze (Spatial Learning and Memory)- The effects of Sex, Age, and Genotype across session on path length to target site in the Morris water maze are presented in Fig. 7A. Overall, path length decreased across session in all groups and path length was higher at 10 and 20 months compared to 5 months. There were no differences between the genotypes at any age. A three-way repeated measures ANOVA revealed significant main effects of Age (p = 0.02) and Session (p < 0.0001) but no other main effects or interactions (all ps > 0.14). The latency to the target site (Fig. 7B) decreased across session in all groups and increased with age at 10 and 20 months. A three-way repeated measures ANOVA revealed significant main effects of Age (p < 0.001) and Session (p < 0.0001) but no other main effects or interactions (all ps > 0.22). Swim speed (Fig. 7C) decreased with age in GCLM^+/+^ mice at 10 and 20 months for both males and females. In GCLM^-/-^ mice, swim speed decreased with age at 10 months in females and at 20 months in both males and females. Swim speed was higher at 10 months in GCLM^-/-^ males compared to GCLM^+/+^ males. A three-way repeated measures ANOVA revealed significant main effects of Age (p = 0.02) and Session (p < 0.0001) but no other main effects or interactions (all ps > 0.14). The effects of Sex, Age, and Genotype on average time spent in the annulus 40 across all sessions are presented in Table 1. Overall, time spent in the annulus 40 in decreased with age at 10 and 20 months. A three-way ANOVA revealed a significant main effect of Age (p < 0.001), but no main effects of Sex or Genotype (ps > 0.10) or significant interactions of Sex, Age, or Genotype (all ps > 0.24). There were no differences between groups on maximum performance (path length average of last two session-maximum performance) in the visible platform test (Table 1) and these observations were supported by the lack of main effects or interactions between Sex, Age and Genotype (all ps > 0.15).

**Figure 9.**
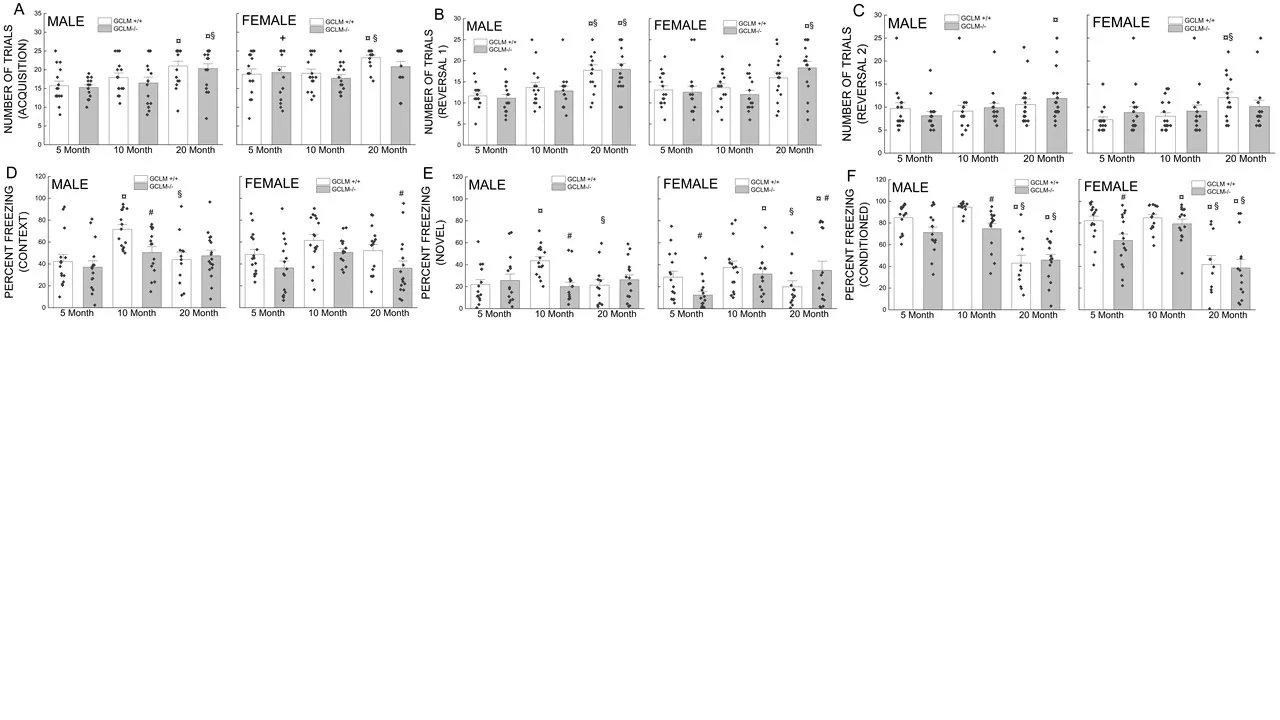
**Effects of Sex, Age and Genotype on cognitive function**. Effects of Sex, Age and Genotype on active avoidance (A-C) and fear response (D-F) in young (5 month), adult (10 month), and old (20 month) GCLM+/+ and GCLM-/- mice. Each value represents the mean ± SEM (n = 12-17). Post-hoc analyses: +*p* < 0.05 compared to age and genotype-matched males; ¤*p* < 0.05 compared to genotype and sex-matched young; §*p* < 0.05 compared to genotype and sex-matched adult; #*p* < 0.05 compared to age and sex-matched GCLM+/+ mice.

Forced Swim Test (depression-like behavior and stress response)- The effects of Sex, Age, and Genotype on immobility time are presented in Fig. 8A. Overall, immobility time decreased with age in all groups, though the pattern of decrease was sex dependent. In males, GCLM^-/-^ had higher immobility time than GCLM^+/+^ at 20 months. A three-way ANOVA revealed a main effect of Age (p < 0.0001), and a significant interaction between Sex*Age (p = 0.01), but no other interactions for Sex, Age, or Genotype (all ps > 0.12).

Auditory Startle (hearing measurement)- The effects of Sex, Age, and Genotype on force production are presented in Fig. 8B. In all groups, force production increased as sound intensity increased. Regardless of genotype, force production was lower in males at 10 and 20 months compared to 5 months, while in females force production was lower only at 20 months. A three-way repeated measures ANOVA revealed significant main effects of Age (p < 0.001) and Session (p < 0.0001), and interactions of Sex*Age (p < 0.01), Age*Session (p < 0.0001), Sex*Age*Session (p < 0.01), and Age*Genotype*Session (p < 0.0001) but no other significant main effects or interactions of Session with Sex, Age, or Genotype (all ps > 0.39).

Discriminative Active Avoidance (learning and cognitive flexibility)- The effects of Sex, Age, and Genotype on the number of trials to reach avoidance criterion are presented in Fig. 9A-C. Overall, it took the 20-month-old mice more trials to reach criterion than the 5- or 10-month-old ones regardless of genotype or session. Overall female mice took more trials to reach criterion during the acquisition phase but less trials during the reversal phases. These observations were supported by a three-way repeated measures ANOVA that revealed significant main effects of Age (p < 0.0001) and Session (p <0.001) and interaction of Sex*Session (p < 0.01) but no other main effects or interactions (all ps > 0.07).

Fear Conditioning (Associative learning)- The effects of Sex, Age, and Genotype on freezing time in the fear conditioning test are presented in Fig. 9E-F. In the context session, context-dependent freezing increased with age especially between 5 and 10 months and more so in male GCLM^+/+^. A three-way ANOVA revealed significant main effects of Age (p < 0.0001) and Genotype (p < 0.01) and no main effect of Sex (p = 0.70) or interactions (all ps > 0.17). In the novel context session, an increase with age was observed in GCLM^+/+^ males (5 vs. 10 months) and in GCLM-/- females (5 vs. 10 and 20 months). These observations were supported by a three-way ANOVA that revealed a significant main effect of Age (p < 0.01) and interactions of Age*Genotype (p < 0.01) and Sex* Age*Genotype (p = 0.03). In the novel context and conditioned stimulus session, freezing declined with age by 20 months. In GCLM^-/-^ mice, 5-month females and 10-month males had lower freezing compared to GCLM^+/+^ counterparts. A three-way ANOVA revealed significant main effects of Age (p < 0.0001) and Genotype (p < 0.01) and an interaction of Age*Genotype approached significance (p = 0.09).

**Figure 10.**
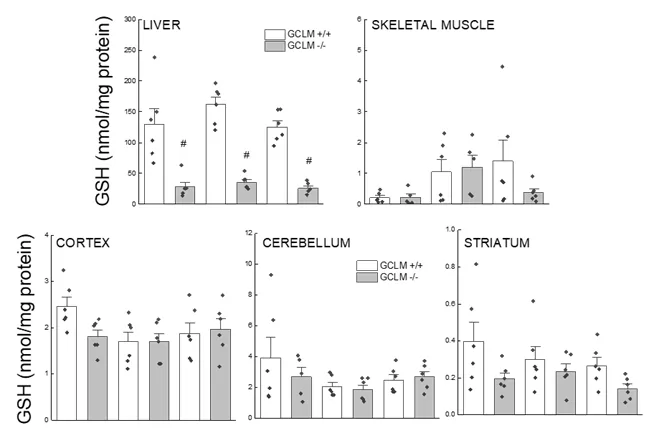
**Effects of Age and Genotype on reduced glutathione levels in tissues**. Effects of Age and Genotype on reduced glutathione levels in the liver, skeletal muscle, cortex, cerebellum, and striatum of young (5 month), adult (10 month), and old (20 month) GCLM+/+ and GCLM-/- mice. Each value represents the mean ± SEM (n = 5-6). Post-hoc analyses; #*p* < 0.05 compared to age-matched GCLM+/+ mice.

Visual Acuity (visual function)- The effects of Sex, Age and Genotype on visual acuity are presented in Table 1. Effects of age were small and most pronounced in the female GCLM^-/-^. These observations were supported by a three-way ANOVA which revealed a significant main effect of Age (p < 0.001) and approached significance for an interaction of Sex*Age*Genotype (p = 0.075).

Glutathione measurements (redox state) - For these measures (and the following ones), we had to combine males and females and were only able to assess the effects of age and genotype. The effects of Age and Genotype on GSH levels are presented in Fig. 10. In the liver, GCLM^-/-^ mice had ~80% lower GSH levels compared to age-matched GCLM^+/+^ mice across ages. Non-parametric analysis revealed a main effect of Group followed by significant pairwise comparisons between genotype at each age (all ps<0.001). None of the analyses for the other tissues yielded significant Group effects though striatum and skeletal muscle approached significance (p= 0.073 and 0.084, respectively).

**Figure 11.**
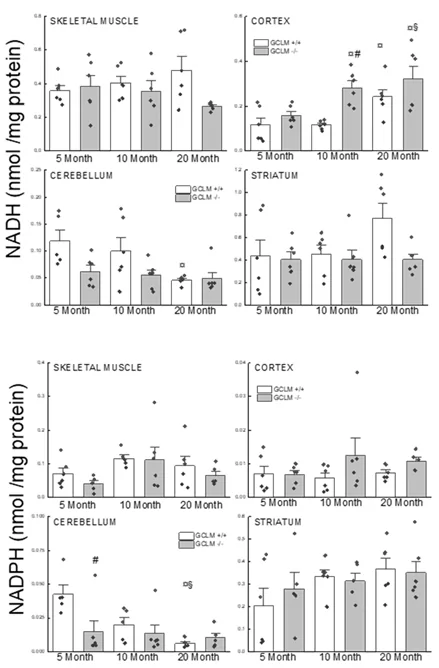
**Effects of Age and Genotype on NADH and NADPH levels in tissues**. Effects of Age and Genotype on NADH and NADPH levels in the skeletal muscle, cortex, cerebellum and striatum in young (5 month), adult (10 month), and old (20 month) GCLM^+/+^ and GCLM^-/-^ mice. Each value represents the mean ± SEM (n = 5-6). Post-hoc analyses; ¤*p* < 0.05 compared to genotype-matched young; § *p* < 0.05 compared to genotype-matched adult; #*p* < 0.05 compared to age-matched GCLM^+/+^ mice.

NADH and NADPH measurements (redox state) - The effects of Age, and Genotype on NADH and NADPH levels are presented in Fig. 11. For NADH, there were no main effects of Group for the muscles and striatum. In the cortex, GCLM^+/+^ mice had higher levels of NADH at 20 months while GCLM^-/-^ mice had higher levels at 10 and 20 months, supported by pairwise comparisons (ps<0.01). There was a genotype effect at 10 months with higher levels of NADH in the GCLM-/- mice (p<0.001). In the cerebellum, GCLM^+/+^ mice had lower levels of NADH at 20 months compared to the 5 and 10 months old. GLCM^-/-^ had lower levels of NADH at 5 and 10 months compared to GLCM^+/+^ but these differences did not reach significance (p>0.059). For NADPH, there were no main effects of Group for the muscles, cortex and striatum. In the cerebellum, NADH levels decreased with age in the GCLM^+/+^ (p<0.05). GCLM-/- mice had lower NADPH levels at 5 months compared to the GCLM^+/+^ mice (p=0.036) and did not change with age.

**Figure 12.**
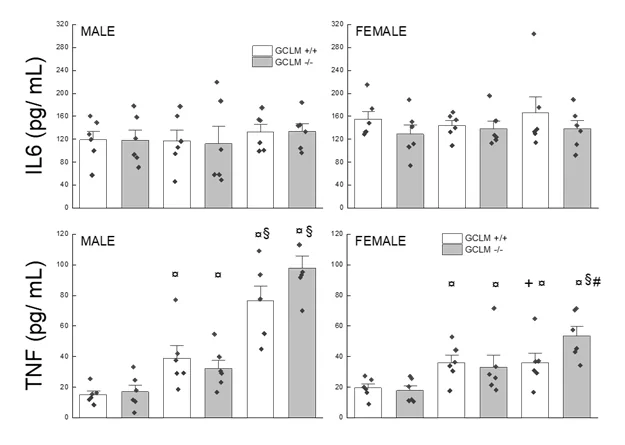
**Effects of Sex, Age and Genotype on IL-6 and TNF-α (B) plasma level**. Values represent the mean ± SEM (n = 6). Pairwise comparisons after significant effect of Group (Kruskal-Wallis): +*p* < 0.05 compared to age and genotype-matched males; ¤*p* < 0.05 compared to genotype and sex-matched young; #*p* < 0.05 compared to age and sex-matched GCLM^+/+^ mice.

Inflammation Measures- The effects of Group on plasma IL-6 and TNF-α are presented in Fig. 12. There were no differences between groups on IL-6 levels (all p=0.800). The levels of TNF were different amongst groups with a significant main effect (p<0.001). In both males and females, we observed a significant increase in TNF levels with age (all ps<0.05). The only effect of genotype was observed at 20 months (p<0.05).

## DISCUSSION

The main findings of the study were (1) GCLM^-/-^ mice had longer lifespans and lower body weights than the GCLM^+/+^; (2) GCLM^-/-^ mice had delayed motor declines; (3) GCLM-/- mice had no major deficits in cognition, but had decreased anxiety-related behaviors and impaired amygdala-dependent memory in 5 and 10 months group; (4) tissue-specific response to knockout of gclm on redox status; (5) sexual dimorphism in motor response; (6) and age-related and sex-dependent increase in TNFα.

The lower body weights of the GCLM^-/-^ mice compared to their GCLM^+/+^ counterparts is primarily attributed to decreased fat tissue rather than lean mass, which is indicative of a lifelong lean phenotype [16]. Other studies in young GCLM^-/-^ mice have reported the same body weight differences, which begin to manifest primarily by 3-4 months of age [16, 17] and maintained throughout the life span [18]. Furthermore GCLM^-/-^ mice have been shown to be resistant to high-fat diet weight gain [16] and diet-induced steatohepatitis [19] by increasing basic metabolic rate and decreasing SCD-1, an enzyme required for triglyceride synthesis [19]. Suppression of SCD-1 has been associated with hypermetabolism and weight loss in mice [19].

Interestingly, this lean phenotype may contribute to the observed increase in lifespan, as GCLM^-/-^ mice exhibited both extended median and maximum lifespans. These findings align with previous studies suggesting that reduced glutathione levels and the resulting oxidative stress may activate compensatory mechanisms that enhance longevity, similar to that observed in caloric restriction models [16, 20, 21]. It was observed that modest decrease in GSH levels, both chemically (Diethyl maleate (DEM)) or with RNAi, extended lifespan and improved resistance to oxidative stress in *C. elegans*. Extension of *C. elegans* lifespan by low-dose DEM was dependent on the presence of transcription factors DAF-16 and SKN-1 (mammalian FOXO and Nrf2) which lead to upregulation of antioxidant genes encoding for antioxidant enzymes such as catalase and superoxide dismutase [20]. Interestingly chronic supplementation of glutathione or its precursor, N-acetyl cysteine, accelerates aging in *C. elegans* through inhibition of skn-1-mediated transcription, whereas a GSH-restricted diet extended lifespan [21]. In addition, methionine (another precursor of GSH) restriction significantly decreases mitochondrial reactive oxygen species production and decreases the electron leak to oxygen in the respiratory chain, thereby reducing steady-state oxidative damage to mitochondrial DNA and proteins [22]. Together, these results suggest that a fine balance of modest decrease glutathione levels is critical for longevity, potentially through mechanisms that involve compensatory oxidative stress response and regulation of key longevity pathways. This highlights the complexity of glutathione’s role in aging, emphasizing the importance of both its physiological levels and the context in which its modulation occurs.

Oxidative stress and damage have been connected with motor function decline in aging rodents [23]. GCLM^-/-^ mice had enhanced motor function, mostly at younger ages, with delayed age-related declines in motor performance though by old age the same performance level was observed as that of GCLM^+/+^. This enhanced function in young mice was independent of changes in body weight, generally was observed in tests assessing balance, coordination, and locomotor activity. This could be due to induction of beneficial adaptations in key brain regions and peripheral tissues involved in motor neuron growth and function by the mild oxidative stress caused by glutathione deficiency. The concept of hormesis, where low to medium levels of stress stimulate adaptive and beneficial responses, may explain in part these observations. For example when a short bout of hypoxia elicits beneficial effects on motor function through growth of motor neuron in the periphery [24]. As intermittent hypoxia induces mild redox stress [24], there may be a common pathway between the present benefits seen in GCLM^-/-^ mice and mice with induced redox stress via hypoxia. We have also previously demonstrated that chronic glutathione deficiency partially and mildly delayed some age-related deficits in gait [25].

While GCLM^-/-^ mice displayed limited impairments in overall cognitive processes, GCLM^-/-^ mice exhibited reduced anxiety-like behavior and impaired fear conditioning, while spatial learning and memory remained intact, consistent with previous studies [26, 27]. GCLM^-/-^ mice were also found to have deficits in pre-pulse inhibition, a phenomenon associated with psychological diseases such as schizophrenia and anxiety-disorders [17]. GCLM has been strongly associated with schizophrenia in some case-control studies [28] with genetic and functional evidence of impairment of GSH synthesis in schizophrenia [29]. Different reasons can be attributed to the selective behavior phenotype seen in the GCLM^-/-^ mice. Chiefly among them is that glutathione deficiency selectively impacts the ventral hippocampus, which is more vulnerable to oxidative stress and is associated with affective behaviors, while sparing the dorsal hippocampus involved in spatial memory [27, 30]. Other studies have also shown changes in brain chemistry in developing GCLM^-/-^ mice, including changes in the anterior cortex similar to clinical data seen in schizophrenia patients [31] Interestingly, Hovatta *et al*., have reported the importance of GSH for regulating these behaviors, where decreased GSH regeneration was associated with decreased anxiety-related behavior [32]. Generally, as rodents age, anxiety-like behavior tends to increase [3] which is also consistent with our findings. However, in our study lifelong glutathione deficiency appeared to attenuate aging-associated increases in anxiety-like behavior. Connecting these neurochemistry deficits in pre-clinical models such as mice to clinical data in humans, suggests a link between impaired GSH metabolism and altered anxiety-related function in both rodents and humans.

Biochemical analyses revealed significant reductions in glutathione levels in the liver, but only sporadic changes in brain regions across the lifespan. Deficits in GSH were seen mainly at 5 months for the striatum, cortex ranging from 20-50%, but the deficits were not consistent across the lifespan and by 10 months there was minimal difference between GCLM^+/+^ and GCLM^-/-^ brain GSH levels. In other studies, the same GCLM^-/-^ mice have shown decreased GSH levels in various brain regions including the cerebellum [33, 34]. However, those studies have focused on young GCLM^-/-^ mice (3-5 months), and it is possible that the GCLM^-/-^ dependent alterations in GSH are age-dependent, as seen in our study where deficits in redox state are most apparent at 5 months. The consistent reduction of GSH in the liver, contrasted with variable reductions in the brain and skeletal muscle, suggests tissue-specific compensatory mechanisms in GCLM^-/-^ mice [35]. The brain may preserve GSH levels through astrocytic synthesis and upregulation of other antioxidants, such as glutaredoxin and thioredoxin, which help mitigate oxidative stress [36]. Additionally, the brain relies on NADPH from the pentose phosphate pathway and other antioxidants like superoxide dismutase and catalase to maintain redox balance [37]. These compensatory mechanisms may explain the minimal cognitive deficits observed in GCLM^-/-^ mice despite GSH depletion. In skeletal muscle, GSH reduction is more sporadic, likely due to its lower oxidative demand and reliance on alternative antioxidant systems, especially during physical stress [38]. Overall, GSH depletion in the liver did not translate to the same depletion levels in the brain which could explain the minimal effects of glutathione deficiency on behavioral outcomes. Additionally, TNF-α, increased with age in both genotypes, more so in females while IL-6 was not affected. These results are consistent with previous studies where TNF-α levels were also increased with age in mice [39] and humans [40]. Inflammation has been linked to GSH for normal brain function, evident in autistic humans who exhibit increased inflammation, lower GSH, higher oxidative damage, and disrupted brain function [41]. Interestingly, young GCLM^-/-^ mice are resistant to hepatic inflammatory fibrosis from a high-sugar diet compared to GCLM^+/+^ mice, showing lower inflammation, oxidative damage, and no steatosis [19]. These mice also display greater resistance to ozone-induced lung injury, with less inflammation due to compensatory antioxidant defenses from chronic GSH deficiency [42]. In human T-cells, GSH depletion impairs NF-kB activation, a key inflammatory mediator, highlighting the relationship between GSH and inflammation regulation [43]. This is confirmed in human liver cells, where GSH depletion limits TNF-α-induced NF-kB activation [44], and in endothelial cells, where GSH depletion inhibits NF-kB activation [45]. Thus, GSH levels modulate NF-kB activation and inflammation in vitro and decreasing GSH levels in vivo in the presents study limits inflammation in young and adult mice but exacerbates it in old GCLM^-/-^ mice. This pattern also corresponds with motor performance in GCLM^-/-^ mice, where young and adult females exhibit better motor skills and decreased inflammation compared to GCLM^+/+^ mice, but worsened performance and increased inflammation in old age. In conclusion, the sex- and age-dependent shifts in motor function in GCLM^-/-^ mice may be partly due to systemic changes in peripheral inflammation. These findings indicate a complex relationship between glutathione deficiency, redox balance, and inflammation, where mild oxidative stress may confer protective effects in younger animals but exacerbate inflammation in older age.

## Conclusions and Limitations

The results of this study challenge the traditional oxidative stress theory of aging by demonstrating that systemic glutathione deficiency does not negatively impact classical health span measures and may extend lifespan [46]. While glutathione is the dominant redox couple in terms of raw substrate, it is one piece in a series of antioxidant enzymatic and redox couples. NADPH/NADP+ for instance, serves as the primary regenerator of GSH/GSSG, and in other studies removing glutathione reductase, the enzyme responsible for this regeneration reaction, results in embryonic lethality [47]. While these mice provide a well-established model for aging and oxidative stress, their physiology, redox responses, and disease progression may not directly translate to humans. Also, mice were kept in highly controlled environmental conditions that may not mimic the complex exposures humans face, such as varying diets and environmental stressors. These findings highlight the importance of considering the nuanced roles of redox state and antioxidant defenses in aging. Further research is needed to elucidate the mechanisms underlying these adaptations and to explore potential therapeutic strategies that leverage mild oxidative stress to promote healthy aging.
